# Effect of Crossing C57BL/6 and FVB Mouse Strains on Basal Cytokine Expression

**DOI:** 10.1155/2015/762419

**Published:** 2015-03-05

**Authors:** Agata Szade, Witold N. Nowak, Krzysztof Szade, Anna Gese, Ryszard Czypicki, Halina Waś, Józef Dulak, Alicja Józkowicz

**Affiliations:** ^1^Department of Medical Biotechnology, Faculty of Biochemistry, Biophysics and Biotechnology, Jagiellonian University, 30-387 Krakow, Poland; ^2^Laboratory of Molecular Neurobiology, Nencki Institute of Experimental Biology, 02-093 Warsaw, Poland; ^3^Malopolska Centre of Biotechnology, Jagiellonian University, 30-387 Krakow, Poland

## Abstract

C57BL/6 is the most often used laboratory mouse strain. However, sometimes it is beneficial to cross the transgenic mice on the C57BL/6 background to the other strain, such as FVB. Although this is a common strategy, the influence of crossing these different strains on homeostatic expression of cytokines is not known. Here we have investigated the differences in the expression of selected cytokines between C57BL/6J and C57BL/6JxFVB mice in serum and skeletal muscle. We have found that only few cytokines were altered by crossing of the strains. Concentrations of IL5, IL7, LIF, MIP-2, and IP-10 were higher in serum of C57BL/6J mice than in C57BL/6JxFVB mice, whereas concentration of G-CSF was lower in C57BL/6J. In the skeletal muscle only the concentration of VEGF was higher in C57BL/6J mice than in C57BL/6JxFVB mice. Concluding, the differences in cytokine expression upon crossing C57BL/6 and FVB strain in basal conditions are not profound.

## 1. Introduction

Laboratory mouse is one of the central model organisms in modern biology, especially since the sequencing of mouse genome. Mouse fanciers and scientists created first inbred strains in the early XX century [1]. Till now numerous mouse types were introduced. Mouse strains differ not only in the coat color but also in blood parameters [2], immune response [3], behavior [4] and reaction to stress [5], and susceptibility to diseases, that is, atherosclerosis [6], diabetes [7] and its complications [8], cancer [9], as well as response to the gene knock-out [10, 11] or transgene expression [12]. Different strains can also vary in lethality after the snake venom treatment [13].

The strain ontology can be assessed with the analysis of single nucleotide polymorphisms (SNP) [14] combined with quantitative trait locus mapping (QTL) and gene expression analysis [15, 16], as well as with microarray gene expression profiling [17] or mitochondrial DNA analysis [18]. Of note, gene targeting by homologous recombination or generation of multiple gene knock-out animals often requires the crossing of different inbred strains of mice and therefore increases mouse genetic variability. However, it may also lead to the phenomenon called hybrid vigor [19] or heterosis [20], which consists in the higher viability and improved parameters of heterozygous progeny in comparison to the inbred parents.

One of the examples where crossing different strains turned out to be beneficial was observed in case heme oxygenase knock-out animals (*Hmox1* ^−/−^).* Hmox1* ^−*/*−^ mice were created on the background of C57BL/6J mice by Poss and Tonegawa [21].* Hmox1* ^−*/*−^ animals were smaller than their wild type littermates, had microcytic anemia, reduced serum iron levels while iron accumulated in kidney and liver, and showed chronic inflammation and splenomegaly [21]. What is more,* Hmox1* ^*+/*−^ mice bred poorly and only less than 5% of newborns from* Hmox1* ^*+/*−^ parents were* Hmox1* ^−*/*−^ homozygotes [21]. Other group reported even lower yield (1-2* Hmox1* ^−*/*−^ homozygotes in 160–200 pups) [22]. Therefore, in order to improve* Hmox1* ^−*/+*^ mice breeding (*Hmox1* ^−/−^ female homozygotes are infertile),* Hmox1* ^*+/*−^ mice were backcrossed 4-5 times to wild type FVB mice. Such* Hmox1* ^−*/*−^ C57BL/6JxFVB mice could be more easily bred, but maintained other characteristics of original* Hmox1* ^−*/*−^ strain [22]. On the other side, the backcrossing of predominantly C57BL/6J to FVB mice resulted as well in creation of another wild type control strain.

Expression of* Hmox1* can be driven by nuclear factor (erythroid-derived 2)-like 2, also known as NFE2L2 or Nrf2, in response to oxidative stress [23]. Nrf2^−/−^ mice were generated in 1997 on the ICR mouse strain background [24] and then backcrossed to C57BL/6 strain for at least 6 generations [25].* Hmox1* ^−*/*−^ mice show impaired regeneration in response to the hind limb ischemia [26] while Nrf2^−/−^ mice show better revascularization due to the inflammatory angiogenesis [27]. However, due to the different genetic background it is difficult to compare directly effects of Hmox1 and Nrf2 deletion. Given that Nrf2/HO-1 pathway is recognized as potent anti-inflammatory mechanism, we were wondering how crossing of C57BL/6 mice with FVB strain expression of the growth factors and inflammatory mediators. This is of particular importance for interpretation of results obtained on mouse with mixed C57BL/6xFVB genetic background that is often the case for mouse gene knock-out models.

## 2. Methods

Animals were handled in a strict accordance with good animal practice as defined by the relevant national and local animal welfare bodies. All animal work was approved by the Local Ethical Committee for Animal Research at the Jagiellonian University. Breeding heterozygote pairs of* Hmox1* deficient mice were initially kindly provided by Dr. Anupam Agarwal, University of Alabama, Birmingham, USA.

### 2.1. Serum Analysis

Female 5-month-old C57BL/6J and C57BL/6JxFVB mice were euthanized by overdosage of xylazine and ketamine. Blood was collected from the vena cava and after clot formation centrifuged for 10 minutes at 1000 g. Serum was collected and frozen at −80°C for further analysis.

Luminex assay (MILLIPLEX MAP Mouse Cytokine/Chemokine-Premixed 32 Plex, Merck, Millipore) was performed according to manufacturer's instructions. The samples were diluted 1 : 1 in Assay Buffer and incubated with Premixed Beads overnight at 4°C. Signal detection was done using FLEXMAP 3D system.

### 2.2. Muscle Lysate Analysis

Female and male C57BL/6J and female C57BL/6JxFVB 2- to 3-month-old mice were injected with 25 *μ*L saline intramuscularly (to both legs). Mice were euthanized by isoflurane overdose and gastrocnemius muscles were excised and homogenized in lysis buffer (PBS, 10% Triton, protease inhibitor cocktail tablets, Roche–1 tablet/50 mL) in tissue lyser (Qiagen).

Luminex assay (The Cytokine Mouse Magnetic 20-Plex Panel, Invitrogen) was performed according to manufacturer's instructions. The samples were diluted in lysis buffer and incubated with Premixed Beads overnight at 4°C. Signal detection was done using FLEXMAP 3D system. Total protein concentration was measured by BCA assay. Briefly, 100 *μ*L of 1 : 50 mixture of CuSO_4_ and bicinchonic acid was added to 5 *μ*L of sample on 96-well plate, incubated 30 min at 37°C and absorbance was measured at 562 nm on Tecan microplate reader.

### 2.3. Statistical Analysis

Statistical analysis was done with GraphPad Prism software. Data are presented as mean + SEM of at least 4 measurements. Two-tailed unpaired *t*-test was applied. Results were considered as statistically significant, when *P* ≤ 0.05 (^*^ *P* ≤ 0.05; ^**^ *P* ≤ 0.01).

## 3. Results

**Figure 1:**
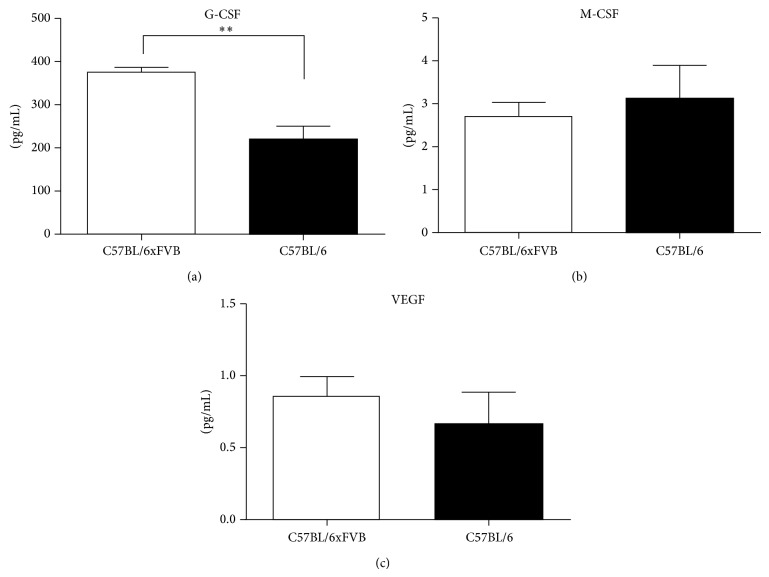
Concentrations of growth factors in serum. G-CSF concentration was higher in C57BL/6JxFVB than in C57BL/6J mice. There were no differences in concentrations of M-CSF and VEGF.

We observed increased concentration of granulocyte colony-stimulating factor (G-CSF) in the serum of C57BL/6JxFVB mice in comparison to C57BL/6J mice (374.5 ± 10.73 pg/mL versus 220.8 pg/mL ± 27.41, *P* = 0.0021, Figure 1). Concentrations of other hematopoietic growth factor, macrophage colony-stimulating factor (M-CSF) was low (<4 pg/mL) and similar between the two strains. The level of granulocyte-macrophage colony-stimulating factor (GM-CSF) was not detectable in the serum. Crossbreeding did not affect the concentration of vascular endothelial growth factor (VEGF) in serum (Figure 1).

**Figure 2:**
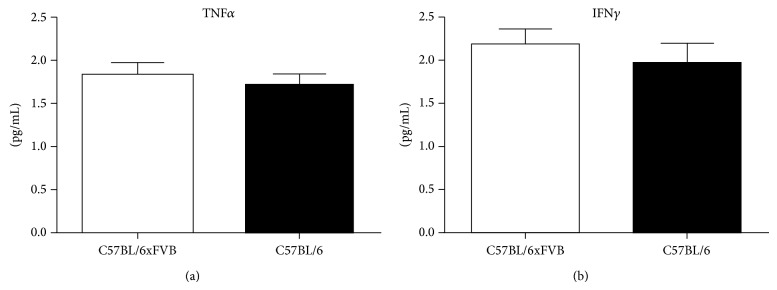
Concentrations of proinflammatory cytokines in serum. Concentrations of TNF*α* and INF*γ* did not differ between C57BL/6JxFVB and C57BL/6J mice.

We observed the same small amounts (~2 pg/mL) of proinflammatory cytokines, tumor necrosis factor *α* (TNF*α*) and interferon *γ* (INF*γ*) in C57BL/6JxFVB and C57BL/6J (Figure 2).

**Figure 3:**
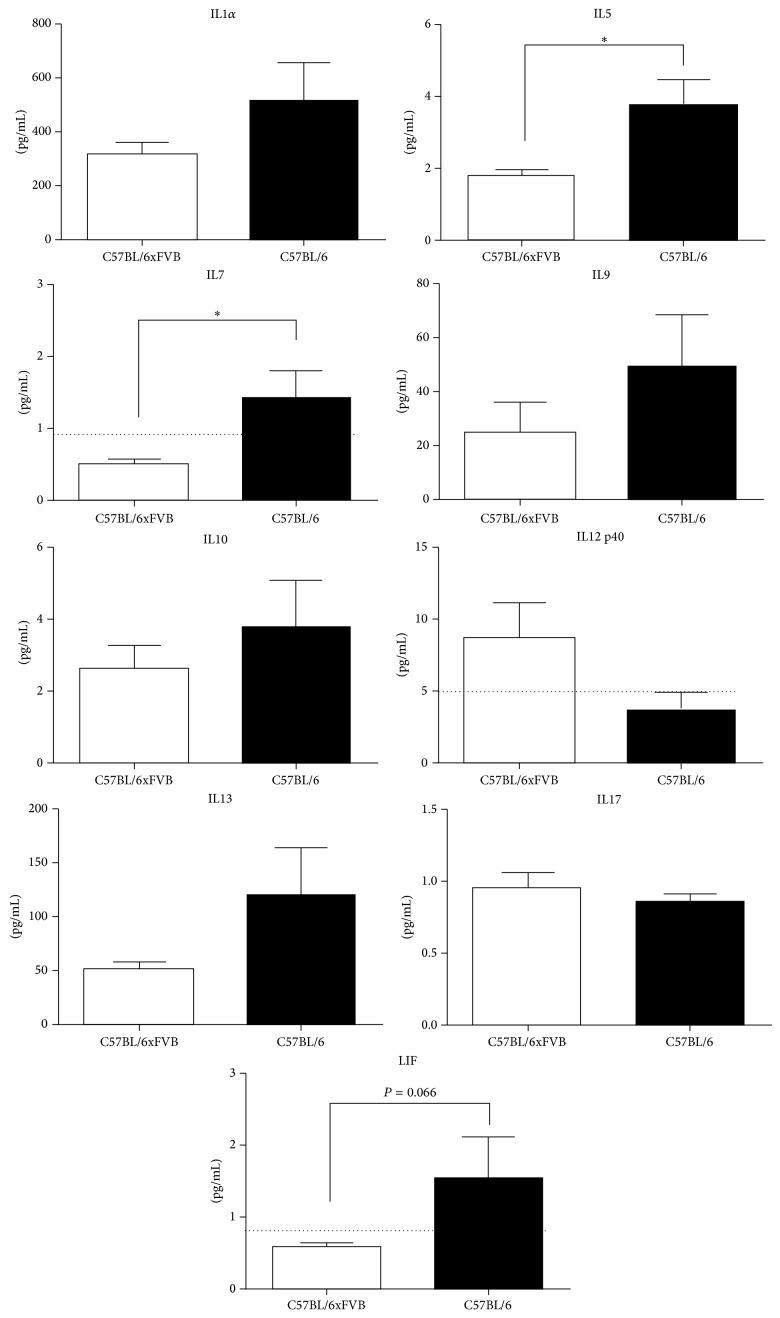
Concentrations of interleukins in serum. Concentrations of IL5, IL7, and LIF were higher in C57BL/6J mice than in C57BL/6JxFVB mice. Concentrations of IL-1*α*, IL9, IL10, IL12p40, IL13, and IL17 did not differ between C57BL/6J and C57BL/6JxFVB mice. Dashed line indicates the detection limit.

Out of the whole panel of interleukins measured, only few were in different concentrations between the strains. IL5, leukemia inhibitory factor (LIF) and IL7 concentrations were lower in the serum of C57BL/6JxFVB than in C57BL/6J mice (Figure 3), however the concentrations of IL7 and LIF were on the border of detection in C57BL/6JxFVB. Concentrations of IL-1*α*, IL9, IL10, IL12p40, IL13 and IL17 were similar in both strains, whereas concentrations of IL1*β*, IL2, IL3, IL4, IL6, IL12p70 were below the detection limit. Most of the interleukins were at levels of 1–10 pg/mL, whereas IL1*α* and IL13 were more abundant (300–500 pg/mL and 50–120 pg/mL, resp.).

**Figure 4:**
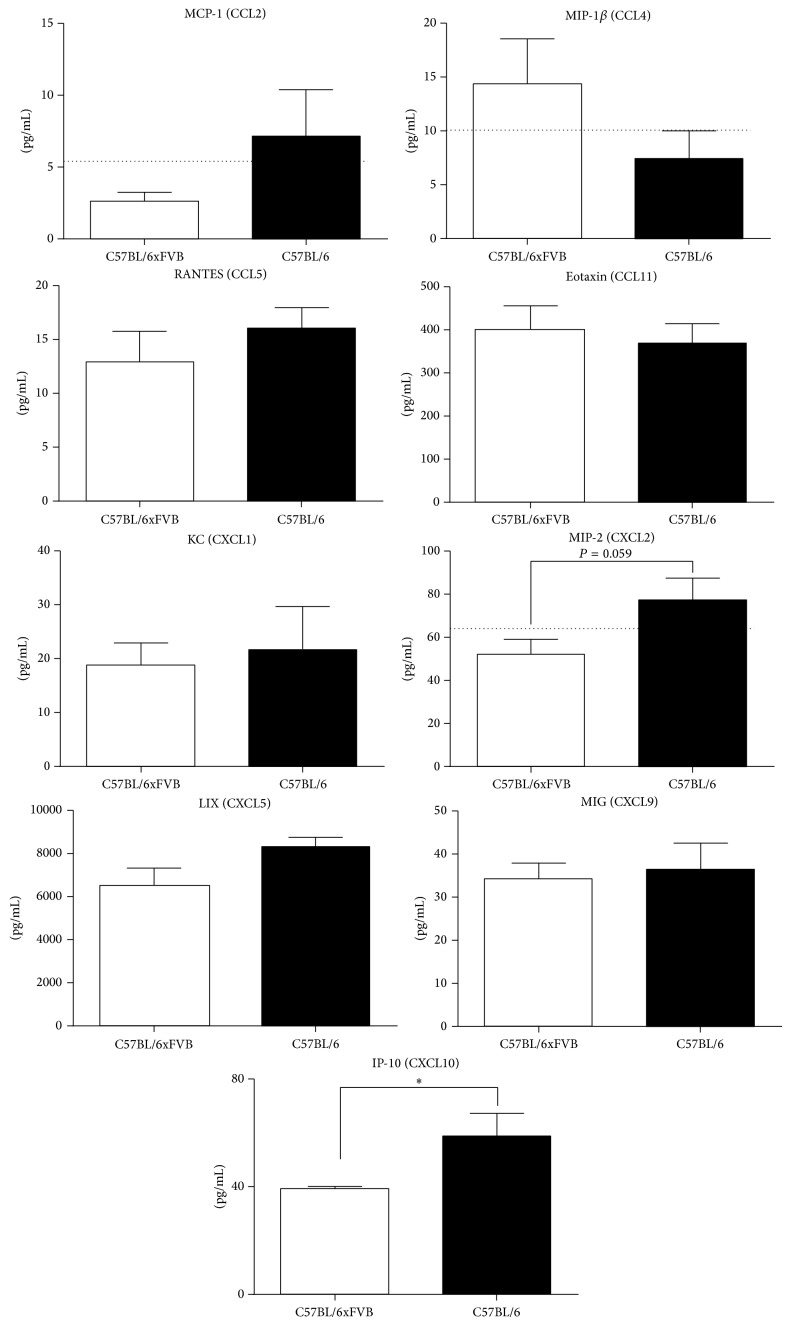
Concentrations of chemokines in serum. Concentrations of MIP-2 and IP-10 were higher in C57BL/6J mice than in C57BL/6JxFVB mice. Concentrations of MCP-1, MIP-1*β*, RANTES, eotaxin, KC, LIX, and MIG did not differ between C57BL/6J and C57BL/6JxFVB mice. Dashed line indicates the detection limit.

Next we have analyzed the concentration of selected chemokines in serum. None of the measured chemokines from the CCL family, monocyte chemotactic protein 1 (MCP-1), macrophage inflammatory protein-1 *β* (MIP-1*β*), RANTES, or eotaxin was different between the compared strains. However two CXCL chemokines, macrophage inflammatory protein-2 (MIP-2) and interferon gamma-induced protein (IP-10), were decreased in serum of C57BL/6JxFVB when compared to C57BL/6J mice (52.1 versus 77.4 pg/mL and 39.3 versus 58.7 pg/mL, resp.). Concentrations of other CXCL chemokines, KC, LPS-induced CXC chemokine (LIX) and monokine induced by gamma interferon (MIG), were the same in both strains (Figure 4). Macrophage inflammatory protein-1 *α* (MIP-1*α*) was not detectable in any of the strains.

**Figure 5:**
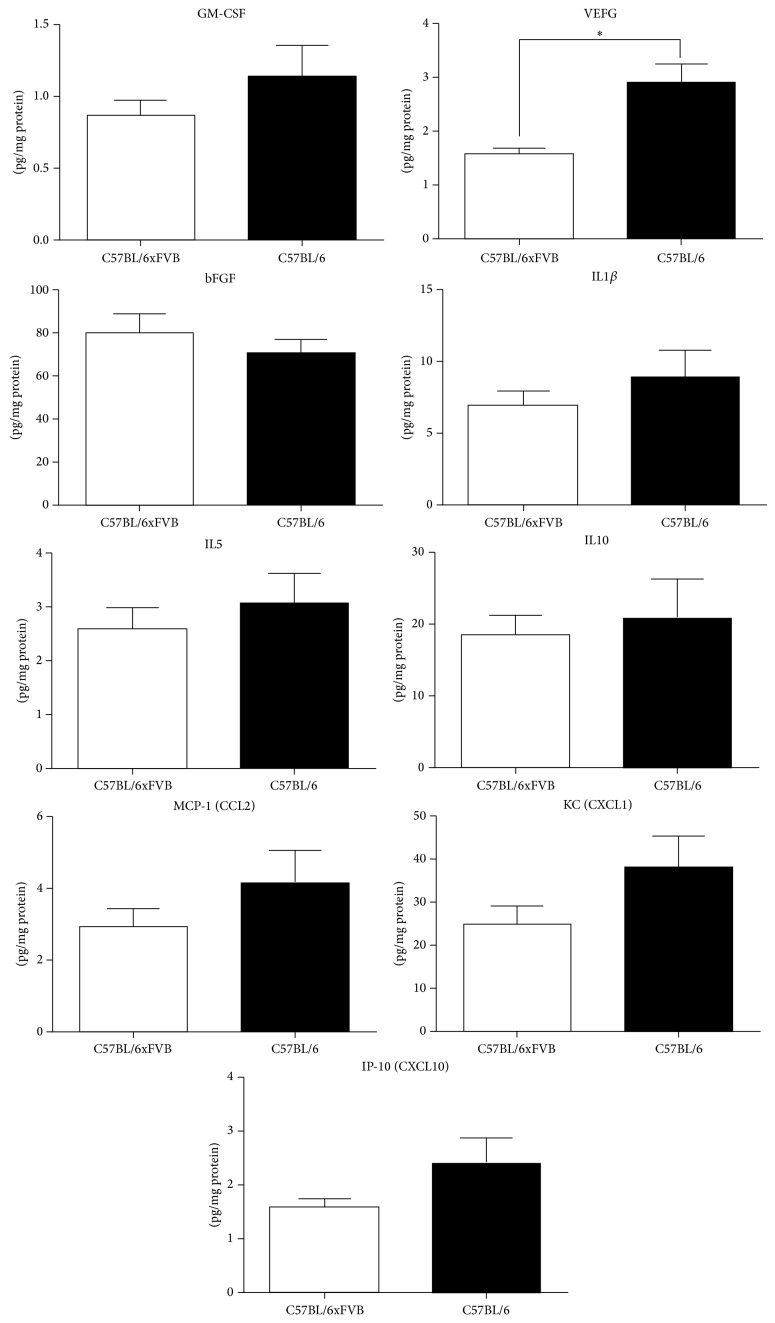
Concentrations of selected cytokines in gastrocnemius muscle. Concentration of VEGF was higher in C57BL/6J mice than in C57BL/6JxFVB mice. Concentrations of GM-CSF, bFGF, IL-1*α*, IL5, IL10, MCP-1, KC, and IP-10 did not differ between C57BL/6J and C57BL/6JxFVB mice.

Finally, we evaluated the expression of several cytokines in the lysates from the gastrocnemius muscle. Cytokines which were in different concentrations in plasma in C57BL/6JxFVB and C57BL/6J, IL5 and IP-10, were at the same concentration in the muscle in both strains. We observed no differences in the concentrations of GM-CSF, IL1b, IL10, MCP-1 and KC in the muscles of C57BL/6JxFVB and C57BL/6J. However, the expression of vascular endothelial growth factor (VEGF) was lower in C57BL/6JxFVB than in C57BL/6J. At the same time concentration of another proangiogenic cytokine, basic fibroblast growth factor (bFGF), was unchanged (Figure 5).

## 4. Discussion

The presented study revealed the degree of changes within concentration of inflammatory mediators in serum and muscles between C57BL/6J strain and mixed C57BL/6JxFVB strain.

It is widely accepted that various mouse strains are phenotypically different. The differences between commonly used mouse strains are also visible on genomic level. Significant gene content variation characterizes the genomes of inbred mouse strains [28, 29] and includes large deletions and amplifications observed between strains. Therefore, when interpreting experimental results one should take into account a possible influence of genetic background of studied mouse strain. This is especially important when choosing controls for knock-out genetic mouse model.

Several phenotypical differences were observed between C57BL/6 and FVB strains. C57BL/6 mice are more susceptible to anesthetic agents, pentobarbital, ketamine, and nitrous oxide in comparison to FVB mice [30]. Mammary tumors develop later in MMTV-PyMT model on C57BL/6J background than on FVB/NJ background [31]. Osteoblasts isolated from C57BL/6 and C3H/HeJ male mice had higher expression and activity of alkaline phosphatase than osteoblasts isolated from female mice, but such difference was not observed in FVB and BALB/c strains [32].

Differences in expression of cytokines between the strains were also reported. For example, profiles of serum cytokines were different both in control mice, as well as after induction of experimental immune thrombocytopenia between C57BL/6J and BALB/c [33].

C57BL/6 strain is commonly used for generation of novel knock-out animal models. However, this strain possesses features that may be unfavorable in given experimental settings comparing to other strains. FVB/N strain is frequently used as a background for transgenic mouse models due to its high fertility and large litters [34, 35]. Therefore, knock-out animals on C57BL/6 background that are hard to breed are often crossed with FVB/N strain to improve their propagation. Accordingly, poor breeding of C57BL/6* Hmox1* ^+/−^ mice was improved after crossing with FVB [22]. Crossing of* Hmox1* ^+/−^ mice which were predominantly on the C57BL/6 background resulted in* Hmox1* ^−/−^ offspring in the number of 2–6.25% of expected mice from Mendelian ratio [22]. In our laboratory* Hmox1* ^−*/*−^ pups are 5.1% of delivered newborns when crossing C57BL/6JxFVB* Hmox1* heterozygotes, what is in consistence with the original report [22]. We could further increase efficiency of* Hmox1* ^*−/−*^ mouse generation to 20.1% of delivered pups when* Hmox1* ^−/−^ males are crossed with* Hmox1* ^+/−^ females. One should be aware, however, that manipulating the background may influence the phenotype of the transgenic animal. Different phenotype of the same mutation in different strains gives the opportunity to choose the strain which better resembles the human disease. In the model of hereditary inclusion body myopathy (HIBM) the Gne^M712T/+^ transgenic mice of C57BL/6 strain developed severe kidney dysfunction and died within few days after birth [36]. Kidney dysfunction is not observed among HIBM patients with GNE mutations, so the mouse model did not reflect properly the human disease. After crossing with FVB, the Gne^M712T/M712T^ glomerular disease was less pronounced and mice survived longer, retaining skeletal muscle pathology [36].

Despite the fact that crossing genetic mouse models on C57BL/6 background with FVB mice helps to avoid limitations, is it has not been studied how such strategy influence the expression of inflammatory mediators. Given that crossing to FVB strain partially rescued the embryonic lethality of* Hmox1* deficiency and glomerular disease of transgenic Gne^M712T/M712T^ mouse we addressed this question in the presented work.

We found several differences in cytokine concentration in serum between the C57BL/6J and the mixed strain C57BL/6JxFVB. IL5 concentration was lower in C57BL/6JxFVB in comparison to the C57BL/6J. However we have not seen any differences in the levels of the same cytokine family members, GM-CSF and IL3. Out of the common cytokine receptor gamma-chain family, only IL7 was affected, concentrations of IL2, IL4, and IL9 remained unchanged. We observed smaller concentration of LIF in serum of C57BL/6JxFVB than in C57BL/6J mice. Level of IL6, member of the same family as LIF, was similar in both mouse strains. However concentrations of these cytokines are very small, on border of detection limit and therefore they may not have the biological significance. The important difference seems to occur in case of G-CSF. C57BL/6JxFVB mice had 1.7x higher concentration of G-CSF than C57BL/6J mice (374.5 versus 220.8 pg/mL, resp.). G-CSF is important for neutrophil precursors proliferation and differentiation, and mature neutrophils release from the bone marrow to the blood [37, 38]. Difference in G-CSF concentration between the strains can supposably affect granulocyte function and their mobilization in response to injury or infection, as well as mobilization of hematopoietic stem cells (HSC). This could be especially important, as C57BL/6 mice are known to be “poor mobilizers” [39].

In the earlier mentioned work by Valles-Ayoub et al. survival of hereditary inclusion body myopathy Gne^M712T/M712T^ transgenic mice was significantly improved by crossing the original C57BL/6 background with FVB strain [36]. Interestingly, Volpi et al. reported the increased concentration of VEGF-A_165b_ in the muscle biopsies from patients with idiopathic inflammatory myopathies, among them sporadic inclusion body myositis (IBM) [40]. Our results show the lower concentration of VEGF in the muscle of mixed C57BL/6JxFVB strain in comparison to C57BL/6J. This might contribute to the less severe phenotype of the disease in Gne^M712T/M712T^ transgenic mice on the mixed background.

Differential expression of cytokines can influence the results of various* in vivo* experiments. It should be also taken into consideration when the cells isolated from mice are cultured* ex vivo* in the presence of cytokines. Possibly, for cells isolated from some of the strains different concentrations of supplemented cytokines may be needed to induce differentiation or functional activity.

## 5. Conclusions

Considering significant genetic differences between various mouse strains it could be expected that concentration of proinflammatory cytokines in often used C57BL/6JxFVB mixed strain will be also affected. However our study showed that crossbreeding of C57BL/6J with FVB mice did not affect majority of cytokines in serum and skeletal muscle. The only cytokines that were significantly different between C57BL/6J and mixed C57BL/6JxFVB strain include G-CSF concentration in serum and VEGF concentration in gastrocnemius muscle. Therefore findings of the study should be considered when using this mixed background in hematological studies due to different G-CSF concentrations in serum and myopathy and muscle regeneration studies due to the different concentrations of VEGF in muscles.
